# Novel Approaches in the Drug Development and Delivery Systems for Age-Related Macular Degeneration

**DOI:** 10.3390/life13020568

**Published:** 2023-02-17

**Authors:** Himanshu Paliwal, Bhupendra Gopalbhai Prajapati, Teerapol Srichana, Sudarshan Singh, Ravish J. Patel

**Affiliations:** 1 Drug Delivery System Excellence Center, Department of Pharmaceutical Technology, Faculty of Pharmaceutical Sciences, Prince of Songkla University, Hat Yai, Songkhla 90110, Thailand; 2 Department of Pharmaceutics and Pharmaceutical Technology, Faculty of Pharmacy, Shree S. K. Patel College of Pharmaceutical Education & Research, Ganpat University, Kherva, Mehsana 384012, Gujarat, India; 3 Department of Pharmaceutical Sciences, Faculty of Pharmacy, Chiang Mai University, Chiang Mai 50200, Thailand; 4 Ramanbhai Patel College of Pharmacy (RPCP), Charotar University of Science and Technology, Anand 388421, Gujarat, India

**Keywords:** age-related macular degeneration, drug delivery, intravitreal route, anti-VEGF, nanotechnology, sustained delivery

## Abstract

The number of patients with ocular disorders has increased due to contributing factors such as aging populations, environmental changes, smoking, genetic abnormalities, etc. Age-related macular degeneration (AMD) is one of the common ocular disorders which may advance to loss of vision in severe cases. The advanced form of AMD is classified into two types, dry (non-exudative) and wet (exudative) AMD. Although several therapeutic approaches are explored for the management of AMD, no approved therapy can substantially slow down the progression of dry AMD into the later stages. The focus of researchers in recent times has been engaged in developing targeted therapeutic products to halt the progression and maintain or improve vision in individuals diagnosed with AMD. The delivery of anti-VEGF agents using intravitreal therapy has found some success in managing AMD, and novel formulation approaches have been introduced in various studies to potentiate the efficacy. Some of the novel approaches, such as hydrogel, microspheres, polymeric nanoparticles, liposomes, implants, etc. have been discussed. Apart from this, subretinal, suprachoroidal, and port delivery systems have also been investigated for biologics and gene therapies. The unmet potential of approved therapeutic products has contributed to several patent applications in recent years. This review outlines the current treatment options, outcomes of recent research studies, and patent details around the novel drug delivery approach for the treatment of AMD.

## 1. Introduction

Age-related macular degeneration (AMD) is one of the common causes of irreversible loss of vision in individuals above 65 years [1,2]. According to the World Health Organization (WHO), the number of AMD cases at present has increased steadily to 196 million, which is expected to accumulate to about 288 million by 2040 [3,4]. It contributes to about 10% of blindness throughout the world by hampering the regular functions of photoreceptors, the retinal pigment epithelium (RPE), and the choroid [5]. Age is the main contributing factor, as people over 85 years of age are ten times more susceptible to AMD [6]. Along with this, genetic background, environmental factors, smoking, para-inflammation, etc. are other important factors [6,7]. However the adoption of a healthy lifestyle for most of the patients with AMD could be useful, which is even more beneficial for patients with a high genetic predisposition. The risk of progression to late-stage AMD can be reduced by half by incorporating an intake of vegetables, fruits, fish, etc. in the diet [8,9]. Quitting smoking is another lifestyle change to combat AMD because active smokers are susceptible to AMD at a rate two to three times that of non-smokers [10]. The risk of genetic factors in AMD is more important in younger individuals. Examples would be variations in genes involved with the complement system and histocompatibility locus antigen (HLA) genes [11,12]. The contribution of other factors such as cardiovascular conditions, exposure to sunlight, alcohol consumption, etc. in AMD progression is unclear [13].

**Figure 1:**
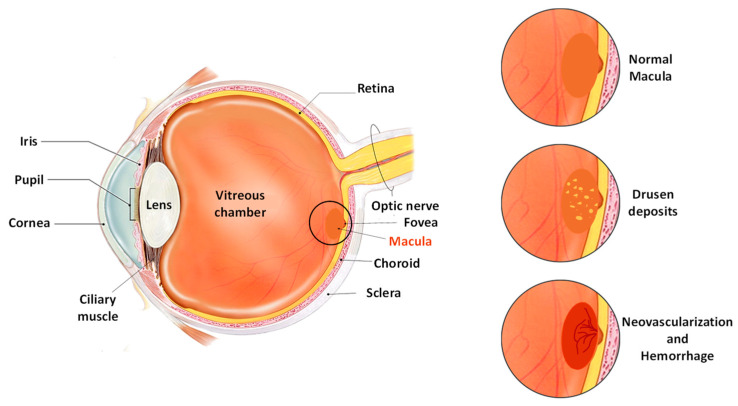
Pictorial representation of the anatomy of the eye in individuals with AMD.

Based on pathophysiologic features, AMD is majorly classified into dry and wet-type. The initial presentation of the disease is the dry or non-exudative type, which may further advance in the later stage into wet or neovascular AMD [14,15]. Although dry AMD accounts for about 90% of overall cases and wet AMD is responsible for only about 10% of cases, severe loss of vision is associated with the latter form [16]. The clinical feature of the disease involves distinct drusen accumulations and pigment vicissitudes in the early stages, leading to geographic atrophy and neovascularization in the later stages of AMD [17]. Drusen are characteristic epithelial deposits composed of lipids, proteins, etc. that are formed as tiny deposits in yellow color [18]. Typically, drusen are found between the basal membrane of RPE and the inner collagenous region of Bruch’s membrane (Figure 1). The occurrence of drusen in the macula marks the incidence of age-related macular degeneration; the size and area of the drusen may also indicate the progression of AMD in the advanced stage [19,20]. The types of drusen in the macula of AMD patients include the hard and discrete type or the soft and diffusive type. The soft and diffusive drusen are considered to be more pathogenic and tend towards choroidal neovascularization (CNV) [21]. Geographic atrophy (GA) is merged regions formed due to dead RPE cells which are covered by the atrophic photoreceptor. It appears initially in the parafoveal region and may later progress into the foveal regions [22]. CNV involves the formation of newer blood vessels around the RPE, or may infiltrate subretinal space. It mostly contributes to impaired and leaking vessels, followed by the accumulation of blood and fluid in the macula [20,23].

The pathologic mechanisms involved in the development and progression of AMD are mainly related to the impairment and degeneration of RPE [24]. The rise in oxidative load and dysfunction of defensive antioxidant mechanisms has also been recognized as one of the key risk factors affecting the progression of the disease. The oxidative degradation with the advancement of age results in the anatomical degeneration of choriocapillaris, subsequently contributing to the reduction in supply of blood to the RPE and photoreceptors [5,25]. The impeded circulation decreases the usual elimination of lipids, proteins, and other byproducts, which accumulate in the form of drusen [18,21]. These accumulations induce the reformation of the extracellular matrix and incite an inflammatory reaction. Owing to the intertwining of such intricate pathologic processes, AMD progresses to atrophy or neovascularization [26,27]. 

Apart from the difficulty in developing novel therapeutic agents due to the complicated pathophysiologic processes involved in the disease progression, the currently available therapies are limited due to safety, efficacy, and delivery issues [28]. AMD affects the posterior segment of the eye where drugs need to pass through morphological and physiological barriers to be therapeutically effective. Moreover, the conventional therapies used for the disease may be troublesome, as they contribute to severe ocular complications, such as conjunctival hemorrhage, detached retina, a rise in intraocular pressure, endophthalmitis, etc [29,30]. Numerous studies have been undertaken with the goal of developing safe and efficacious products for the management of AMD and to assess their viability through preclinical and clinical investigations [31]. In recent times, the main focus of the researchers has been on novel long-acting approaches with increased targetability to the affected sites and reduced side effects [32,33]. 

## 2. Current Approaches for the Treatment of AMD

**Figure 2:**
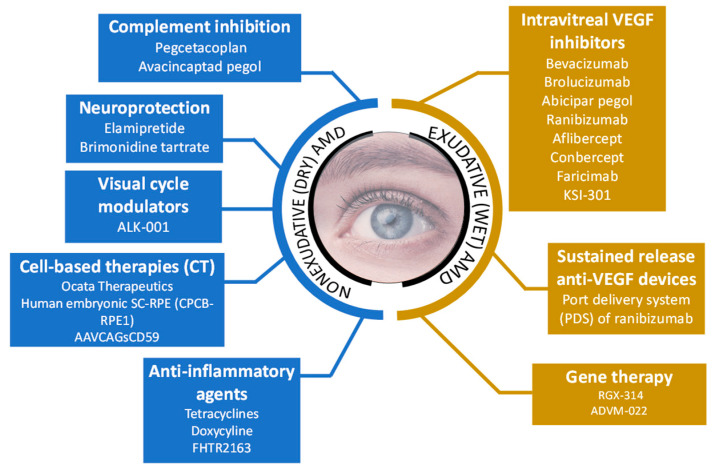
Illustration of approaches used by currently available therapies for non-exudative (dry) AMD and exudative (wet) AMD along with some of the examples of clinically investigated drugs [78].

Several attempts have been made to develop reliable therapeutic and diagnostic tools for the effective management of AMD. The investigated therapeutic approaches as depicted in Figure 2 are mostly focused on the prevention of disease, slowing down disease progression, and restoring impaired vision [34]. The treatment approaches for dry and wet-type AMD along with some important examples are discussed as follows:

### 2.1. Inhibition of Complement System

The inhibition of the complement system is one of the approaches which involves the suppression of complement proteins to downregulate the complementary pathways and the formation of the membrane attack complex [35]. The complement system is usually triggered through three pathways: the classical pathway, the lectin pathway, and the alternative pathway. The classical and lectin pathways recognize the polysaccharides or glycoproteins on the impaired surface of the cell to generate C3 convertase (C4b2a) [36]. C3 is one of the major complement proteins which are cleaved by C3 convertase, generating C3a and C3b proteins. Additionally, the alternative pathway has positive feedback on the production of higher amounts of C3b [37]. Consequently, the C3b generation contributes to the formation of C5 convertase, which further splits C5 into C5a and C5b. This is followed by employing C5, C7, C8, and C8 to induce the formation of a membrane attack complex (C5b-9). The terminal complex; C5b-9, is responsible for creating pores in the cell membrane leading to the lysis and death of the cell [38,39]. Pegcetacoplan (APL-2) is a PEGylated peptide molecule and selective inhibitor of C3 used against GA pre- and post-AMD [40]. A C5 targeting product; ARC1905 (ongoing clinical trial Phase III) was reported to have adequate safety and tolerability, and as showed a 27% decrease in GA lesions [41]. IONIS-FB-LRx, an anti-sense oligonucleotide that suppresses the alternative pathway, was reported to show a considerable decrease in circulating complement factor B, which has a high affinity for C3b. This results in a reduction in the generation of alternative C3 convertase and GA lesions [42]. Factor H is another major complement factor that competes with factor B for binding with C3b. In addition, factor H also binds to C-reactive protein (CRP) and amplifies the complement inhibition, especially in cases of impaired or apoptotic cells due to inflammatory reactions. However, a group of protein called complement factor H-related (CFHR) protein regulates the interaction of FH with different cells. The C-terminal homology of CFHR with FH leads to competitive binding with C3b to evade the FH activity and augment the activation of the completement system [43].

### 2.2. Modulation of the Visual Cycle

In AMD patients, the visual cycle involving enzymatic reactions in the RPE and photoreceptor cells of the retina along with phototransduction cascade events may produce significant inflammatory and metabolic waste. The progressive build-up of these wastes culminates in GA and retinal impairment [24]. Visual cycle modulators may be incorporated to slow down the visual cycle, thereby reducing the accumulation of these byproducts. The mechanism of action of these modulators includes supplementing with deuterated vitamin A, suppression of visual cycle enzymes, the inhibition of retinol-binding protein 4 (RBP4), and scavenging free all-trans-RAL [44]. Visual transduction is coordinated by vitamin A in the body, but the higher accumulation of vitamin A dimers is reported to be associated with the development of AMD [45]. A synthetic deuterated vitamin A formulation (ALK-001) was proposed for replacing the vitamin A in the body and slowing down the disease progression [46]. 

### 2.3. Cell-Based Therapies

The transplantation or implantation of the cell can be an alternative therapy in AMD. Although a substantial number of cells of RPE may be damaged in AMD patients, other cells such as photoreceptors, ganglion cells, bipolar cells, etc. may sustain efficient retinal connections [47]. Therefore, the replacement of the damaged RPE cells might be a suitable strategy for the treatment of AMD. The replacement of degenerated RPE cells with healthy cell transplants have been shown to restore impaired photoreceptors along with improved vision [48,49]. Stem cell therapy comprises the incorporation of new RPE cells in the subretinal region for restoring the functions of RPE cells, which further improves the functions of photoreceptors [50,51]. The reversal of differentiated cells into the pluripotent stage enabling their revival into other cell types is one of the major approaches to cell-based therapies [51,52]. On the other hand, the non-stem cell approach is purely based on the implantation of cells, which can produce protective factors that are insufficient [53]. A stem cell-based approach involving human embryonic stem cells was reported to show considerable improvement in visual functions and subretinal pigment in the atrophic region [54]. The improvement in visual function was also displayed by the California Project to Cure Blindness-Retinal Pigment Epithelium 1 (CPCB-RPE1), which is a human embryonic stem cell-derived RPE (hESC-RPE) [55].

### 2.4. Reduction of Inflammation

Inflammation has been reported to play a major role in the progression and pathogenesis of AMD [56]. Therefore, the inclusion of drugs in AMD therapy to reduce inflammation has been recommended [57]. Tetracycline possesses anti-inflammatory potential in AMD by averting the complement activation, reducing the production of cytokines and chemokines, etc., which further aids in reducing cell damage [58]. Doxycycline and high-temperature requirement A serine peptidase 1 (HTRA1) gene targeting antibodies were also investigated for the treatment of GA [59,60]. The genetic variation related to 10q26 region of chromosome 10 is one of the major risk factors for AMD. 10q26 is associated with HTRA1 genes. The variants of these HTRA1 regulatory regions may result in the decreased expression of HTRA1 in RPE. Consequently, the reduction in HTRA1 protein within RPE and Bruch’s membrane marks the initiation of AMD. The enhancement of HTRA1 expression may be one of the feasible therapeutic strategies for the treatment of AMD [61]. 

### 2.5. Neuroprotective Strategies

Neuroprotection involves the use of therapeutic agents to enhance neuronal endurance by protecting the anatomical and physiological features of the neurons. The neuroprotective agents in AMD are very important along with other therapeutic strategies for slowing down the progression and preventing the loss of vision [62,63]. Elamipretide, a tetrapeptide-based agent, is effective in decreasing oxidative stress and mitochondrial dysfunction, thereby controlling the disease progression [64]. The use of brimonidine tartrate has also been recommended for the management of GA and associated glaucoma-like conditions for retinal degeneration diseases [65]. 

### 2.6. Inhibition of Vascular Endothelial Growth Factor (VEGF)

The pathogenesis of AMD involves complicated pathways pertaining to fluctuations in angiogenesis and pro-angiogenesis factors, such as; (i) VEGF overexpression, (ii) pigment epithelium-derived factor (PEDF) deficit, (iii) the reduced expression of the extracellular domain of VEGF receptor 1, and (iv) the inhibition of pro-angiogenic factors [66,67]. Treatment of neovascular AMD with humanized monoclonal antibodies, such as bevacizumab and ranibizumab, are reported to be highly effective. Brolucizumab is a humanized antibody fragment administered by the intravitreal route and approved for the treatment of neovascular AMD through the inhibition of VEGF [68]. Abicipar pegol, a newly designed ankyrin repeat protein, is known to bind to and inhibit VEGF A. It is reported to be highly efficacious in controlling neovascularization and retaining vision in 90% of patients [69]. Aflibercept and conbercept can inhibit placental growth factors as well as VEGF-A, VEGF-B, and VEGF-C, exhibiting multiple targets and more efficacy in the treatment of exudative AMD [70]. Bispecific antibody-like faricimab inhibits both angiopoietin-2 and VEGF-A, and possesses superior therapeutic action in reducing vascular leaking and inflammatory responses [71]. 

Despite the high efficacy of intravitreal therapy, it is associated with the burdens of frequent injections, high treatment costs, and is associated with the risk of an increase in intraocular pressure [72]. RGX-314 is a product developed to surgically deliver the AAV8 vector which expresses the monoclonal antibody capable of suppressing VEGF activity [73]. Another gene therapy product, ADVM-022, is developed for attaining the sustained intraocular expression of aflibercept [74]. Both of these products are given as targeted intravitreal injections focusing on inhibiting VEGF signaling, followed by controlling the angiogenic and vascular leakage attributes of wet AMD [73,75].

### 2.7. Sustained Releasing Anti-VEGF Devices

The delivery devices of anti-VEGF based on the port delivery system allows for their sustained release into the eyes. Ranibizumab (anti-VEGF antibody fragment) has been formulated and encased in sustained-release devices. The devices were able to achieve similar treatment outcomes with a considerably reduced frequency of administration [76,77].

## 3. Novel Drug Delivery Systems for AMD

**Figure 3:**
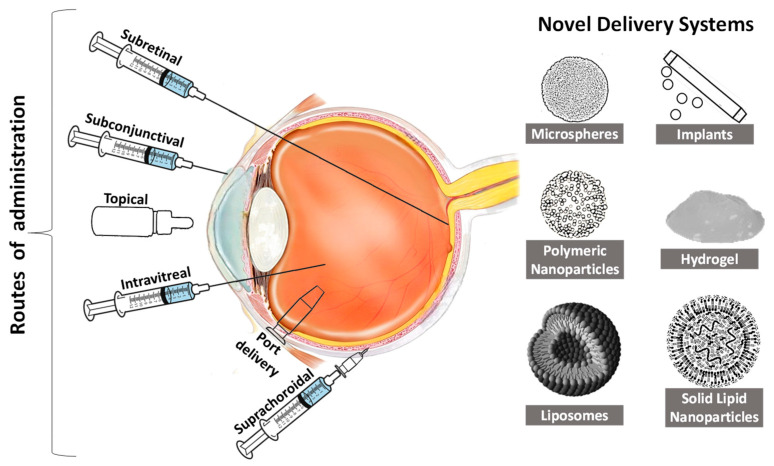
Routes of administration for the ocular drug delivery system for age-related macular degeneration.

The delivery systems used for therapeutic agents against AMD primarily revolve around using either implantable devices or intravitreal injectable liquids. Recently, scientists have made considerable attempts at improving the potential of therapeutic agents used for AMD by designing novel carriers that can reduce invasiveness at a low cost [31,79]. Despite technological developments achieved for the delivery of ophthalmic agents, there is still a need for a more reliable approach owing to the bioavailability and penetrability issues presented due to ocular barriers. In the case of reduced bioavailability, the viability of therapeutic agents for AMD becomes hampered, as they require frequent administration [80]. The approaches under investigation have attempted to improve the bioavailability of the drugs either by using nanoparticulate carriers [81], sustained-release products [82], or penetration enhancers [83]. This section of the review is focused on a discussion of novel drug delivery systems targeting different routes of administration (Figure 3), with a special emphasis on their applications and limitations.

### 3.1. Intravitreal Route of Delivery

Intravitreal space is the most employed route for the delivery of therapeutic agents for AMD. The main reason behind the popularity of this route is its high safety, minimum invasiveness, convenient application, and adequate efficacy [84]. Table 1 covers the overview of the safety and efficacy of age-related macular degeneration management with various therapies. Macular edema is one of the common features of neovascular or wet AMD, and intravitreal antibodies targeting VEGF have been shown to decrease macular edema to further avoid loss of vision. Therefore, all of the therapies that are intravitreally injected target wet AMD [85]. Control composite microspheres of bevacizumab may be useful in prolonging the drug release to avoid frequent monthly administration (Table 2). The microspheres containing poly(D, L-lactide-co-glycolide)/poly(cyclohexane-1,4-diyl acetone dimethylene ketal) (PLGA/PCADK) were produced using a solid-in-oil-in-water emulsification method. The smooth spherical microspheres showed an initial burst release, followed by a sustained release for about 50 days. Adequate encapsulation efficiency and better tolerability to ocular tissues make these composite microspheres a potential candidate for delivering drugs against ocular diseases [86]. PLGA-based nanoparticles were fabricated to enhance the shelf-life and stability of bevacizumab, along with imparting a controlled release feature. The negative zeta potential (−23.1 mV) and high encapsulation efficiency (82.47%) indicated the adequate stability and efficiency of the nanoparticle formulation. The pH-dependent release of bevacizumab was also observed over 168 h, and the release of the drug was significantly higher at pH 10 than at pH 6 and 7.4. MTT and bromodeoxyuridine (BrdU) proliferation assays showed no significant differences in the bioactivity of bevacizumab when loaded into PLGA nanoparticles [87]. Another attempt of developing PLGA nanoparticles of bevacizumab was carried out to increase its residence time in the aqueous and vitreous humor for prolonging the duration of action. The study reported that nanoparticles demonstrated the enhancement of bioavailability and the anti-VEGF activity of bevacizumab, along with no major signs of cytotoxicity and tissue toxicity. The incorporation of PLGA further improved the anti-angiogenic effect by suppressing corneal and retinal neovascularization [88]. 

The mesoporous silica nanoparticles (MSNs) functionalized with 3-aminopropyltriethoxysilane (3-aminopropyl) triethoxysilane (APTES) and mPEG-succinimidyl carboxymethyl ester (mPEG-NHS) were also reported to show improvement in the anti-VEGF potential of bevacizumab. In vitro studies showed that MSNs were effective in inhibiting proliferation, migration, and tube formation of endothelial cells induced by VEGF. Furthermore, the MSNs exhibited in vivo inhibition of corneal and retinal neovascularization [89]. Bevacizumab containing chitosan nanoparticles was developed using the ionic gelation method meant for embedding in the ocular implant. The homogeneously formed nanoparticles (particle size ~78.5 nm) displayed the extended release of bevacizumab over a 2-month study [90]. The preparation of a chitosan grafted-poly(ethylene glycol) methacrylate-(CS-g-PEGMA) based polymeric nanocarrier of bevacizumab was carried out by the first synthesis of CS-g-PEGMA by the Michael addition reaction, and nanoparticles were designed by the double crosslinking of reverse emulsion. The study reported uniform spherical particles that possess pH-sensitive properties in aqueous conditions. The nanoparticles showed the controlled release of bevacizumab for more than 72 h due to the swelling tendency in aqueous solution. The preparation designed for local injection was found to be completely hemocompatible without any significant toxicity indications [91]. Mu et al. prepared multivesicular liposomes of bevacizumab by the double emulsification technique and the use of 10% human serum albumin preserved the activity of bevacizumab for a longer duration. The bevacizumab was released from the liposomes in a sustained fashion for up to 14 days, owing to slow erosion and diffusion from the vesicles. The lesions of CNV were reduced in the rats after 28 days of treatment with bevacizumab containing liposomes due to the prolonged retention in the vitreous humor [92].

Vollrath et al. developed sustained-release solid lipid implants of ranibizumab using the twin-screw extrusion method. The implant could load a high amount of protein (3 mg/implant) with consistently sustained release profiles for 120 days. The implant showed a predominantly high release of the monomeric form (>95%) of the ranibizumab initially, followed by the formation of the hydrophobic type upon the completion of 18 weeks. The stability of the ranibizumab in the implant was exceptional with no signs of aggregation or alterations in secondary structures [93]. PLGA microparticles have been investigated for fabricating sustainable release systems of mABs such as ranibizumab for enhancing their anti-angiogenic potential and protection against proteolytic degradation. The microparticles sustained the release of ranibizumab, and about 80% release was achieved after the completion of 3 weeks. The cell proliferation and tube formation assay of the formulation showed a considerable reduction in VEGF-induced tube formation [94]. PLGA-based microspheres of ranibizumab showed a significantly greater reduction in lesions of CNV in studied animals at lower doses. Apart from the mild and insignificant rise in intraocular pressure, there was no signal of cellular dysfunction of the retina in the electroretinogram [95]. The biodegradable microsphere-based hydrogel of ranibizumab was designed by Liu et al. to impart controlled release characteristics. Volume phase transition temperature (VPTT) showed an increase while swelling ratios decreased with the corresponding increment in the concentration of a cross-linker. The microspheres demonstrated the pH-sensitive controlled release of ranibizumab for up to 6 months, and relatively rapid release can be attained by increasing the concentration of the degradable cross-linking agent [96]. 

The researchers used a similar delivery system for the encapsulation of aflibercept. The rate and extent of release of aflibercept was also in a controlled manner depending upon the concentration of the cross-linking agent and the loaded microspheres. The drug released from the microspheres showed no signs of cytotoxicity from its degraded byproducts, and bioactivity was maintained throughout the complete release period [97]. The prepared formulation was injected into the rhesus macaques and observed in the vitreous fluid for 6 months after injection. There were no signs of alterations in the anatomy and physiology of the retina, along with the observation of about 2.1 ng/µL of aflibercept in the vitreous [98]. Polymeric nanoparticles of aflibercept also depicted the sustained release of the protein over 7 days. The nanoparticles showed uniform distribution with insignificant signs of toxicity in the ARPE-19 cells [99]. Adamson et al. reported the production and characterization of microparticles of PolyActive™ hydrogel co-polymer. The outcomes of the study demonstrated the sustained delivery of domain antibodies from microparticles in the rabbit and primate eyes for 6 months. The amount of anti-VEGF agents released in the ocular regions was enough to assert the protection of cynomolgus against laser-induced grade IV CNV (Table 2) [100]. A sustained release intravitreal implant of dexamethasone was prepared, and drug release behavior was studied using various dissolution conditions and methods, such as the shaking incubator experiment, the EyeMovement System (EyeMoS), the USP apparatus 7, and the Vitreous Model. The outcomes of the drug release from different test media, apparatus, and methods displayed high variation. Furthermore, the models and conditions were only able to depict the release of the drug from a gelled compartment, and none of the techniques were able to adequately predict the in vivo performance of the implants [101]. Table 2 illustrates the outcomes of some recent studies based on the intravitreal delivery of novel formulations for the treatment of AMD.

**Table 1:** Summarized safety and efficacy of age-related macular degeneration management with various therapies.

Study Objective	Study Design	Type of Therapy Involved	Outcome with Safety Reports	References
Assessment of safety and efficacy of verteporfin therapy in AMD patients from Japan	Fifty-year-old patients with best-corrected visual acuity, classic-counting choroidal neovascularization secondary to AMD, and a lesion of greater linear dimension ≤5400 µm.	Treated with verteporfin intravenously followed with the administration of light for 15 min	Verteporfin therapy for choroidal neovascularization due to AMD demonstrated similar safety and an effective angiographic and vision effect as that reported in Caucasian patients.	[102]
Safety and efficacy assessment of verteporfin photodynamic therapy in sub-foveal choroidal neovascularization (CNV) related to AMD	Forty-eight patients with sub-foveal CNV secondary to AMD were enrolled with follow-up for at least one year	Treated with verteporfin photodynamic therapy	The study indicated an 83.3% improvement in patients with CNV secondary to AMD with a reduction in visual acuity deterioration due to sub-foveal CNV.	[103]
Efficacy and safety of photodynamic therapy with verteporfin combined with intravitreal triamcinolone in choroidal neovascularization to AMD	One hundred eighty-four patients with sub-foveal choroidal neovascularization with follow-up intervals of 3 months.	A solution containing 25 mg of triamcinolone was injected intravitreally after photodynamic therapy	Pooled verteporfin photodynamic therapy with intravitreal triamcinolone showed significant improvement in efficacy compared to standard verteporfin photodynamic therapy	[104]
Efficacy and safety of combined ranibizumab with verteporfin photodynamic compared with monotherapy of ranibizumab in patients with sub-foveal choroidal neovascularization AMD	Double-masked randomized phase lllb clinical trial on 321 patients	Combination of ranibizumab with verteporfin photodynamic compared with monotherapy of ranibizumab	A well-tolerated result after 12 months of applications was demonstrated in the clinical trial result	[105]
Safety and efficacy after twenty-four-month angiographic outcomes from clinical trials studying photodynamic therapy with verteporfin	Double-masked, placebo-controlled, randomized clinical trial	Patients visiting 28 ophthalmology practices in Europe and North America were treated with verteporfin and placebo-controlled	Verteporfin therapy for sub-foveal choroidal neovascularization caused by pathologic myopia with AMD demonstrated maintenance of safety throughout treatment for 24 months with improved visual acuity.	[106]
Effect of lesion size, visual acuity, and lesion composition on visual acuity change with or without verteporfin therapy	Three placebo-controlled, randomized clinical trials	Treatment of patient (minimally classic lesion with AMD and with no classic choroidal neovascularization with AMD) with verteporfin in photodynamic therapy	Lesion size after treatment of AMD with photodynamic therapy followed with AMD indicated improvement in vision with significant safety and efficacy	[107]
Safety and efficacy of photodynamic therapy combined with verteporfin and bevacizumab	Randomized controlled pilot clinical trial on one hundred and sixty-five patients with classic or occult CNV owing to AMD in at least one eye that had been never been treated previously	Treatment with either single photodynamic therapy with verteporfin or single administration of intravitreal bevacizumab, or in combination	Significant improvement in safety in visual acuity after treatment of one month followed with maintenance over three months was observed	[108]
Comparison of safety and efficacy of anecortave acetate with verteporfin and photodynamic therapy	Five hundred thirty patients with classic sub-foveal choroidal neovascularization with AMD tested following masked, randomized, multicenter, parallel-group, active control, noninferiority clinical trial	Tenon’s capsule-based anecortave acetate periocular posterior juxta scleral depot was administered for six months in the test group, compared with verteporfin photodynamic therapy	The results demonstrated safety and efficacy with the benefits of anecortave acetae for the treatment of choroidal neovascularization risk associated with either the drug or photodynamic therapy	[109]
Visual outcome after the intravitreal injection of triamcinolone acetonide for exudative AMD	Comparative non-randomized clinical trial	Twenty patients with bilateral exudative AMD were treated with a unilateral intravitreal injection of triamcinolone acetonide	Intravitreal injection of triamcinolone acetonide improved the visual acuity of the eye with preconceived safety	[110]
Anti-vascular endothelial growth factor antibody for the treatment of predominantly classic neovascularization in AMD patients	Randomized, double-masked, active-treatment-controlled clinical trial	Treatment of AMD patients with ranibizumab and compared with verteporfin photodynamic therapy	Ranibizumab indicated significant improvement in the verteporfin photodynamic therapy after two years of clinical trials on patients with AMD	[111]
Safety and efficacy assessment of verteporfin photodynamic therapy in reduction of vision loss for the patient with sub-foveal occult associated with AMD	A total of three hundred sixty-four eligible patients with ≥50 years were included in the study	The patients were treated with verteporfin photodynamic therapy and compared with placebo considering the baseline of visual acuity	Verteporfin photodynamic therapy in the management of occult associated with AMD demonstrated safety and efficacy	[112]

**Table 2:** Summary of recent publications (2017 to 2022) of novel drug delivery systems administered through the intravitreal route for the treatment of AMD.

Formulation	Bioactive Agent	Composition	Preparation Method	Significant Outcomes	References
Microspheres	Bevacizumab	PLGA, PCADK, dextran, PEG	Solid-in-oil-in-water emulsification method	Biphasic release pattern; sustained release of about 75.7% within 50 days Insignificant ocular irritation	[86]
Polymeric nanoparticles	Bevacizumab	PLGA and PVA	Modified solvent emulsification-evaporation method	Stable formulation with high encapsulation efficiency pH-dependent release of mAB	[87]
Polymeric nanoparticles	Bevacizumab	PLGA, Tween 80, and PVA	Modified double-emulsion solvent evaporation procedure	Bioavailability enhancement Suppressing neovascularization Prolonged therapeutic efficacy	[88]
Mesoporous silica nanoparticles (MSNs)	Bevacizumab	Tetraethyl orthosilicate (TEOS), cetyltrimethylammonium chloride (CTAC), triethanolamine (TEA), APTES, and mPEG-NHS	Soft template method and Nanocasting strategy	Initial rapid release, followed by sustained release up to 7 days Effective in suppressing VEGF-induced changes in vitro Sustained inhibition of neovascularization in vivo	[89]
Polymeric nanoparticles	Bevacizumab	Chitosan and Sodium tripolyphosphate	Ionic gelation method	Homogeneously dispersed particles with high entrapment efficiency Sustained release for about 2 months	[90]
Modified polymeric nanocarriers	Bevacizumab	Chitosan, Poly(ethylene glycol) methacrylate, Tween 80, Span 80, Sodium tripolyphosphate, and Sodium sulfate	Michael addition reaction and double crosslinking (ionic and covalent) reaction in reverse emulsion	Uniform spherical-shaped nanoparticles pH-responsive controlled release of mABs Hemocompatibility	[91]
Multivesicular liposomes	Bevacizumab	1,2-dioleoyl-sn-glycero-3-phosphocholine, 2-dipalmitoyl-sn-glycero-3-phosphoglycerol, cholesterol, and triolein	Double emulsification technique	Sustained release of bevacizumab for up to 14 days Significant reduction in CNV lesions Biologically feasible and prolonged retention in the vitreous humor	[92]
Solid lipid implants	Ranibizumab	Triglycerides H12, Triglycerides Dynasan D118, and Hydroxypropyl-β-cyclodextrine (HP-β-CD)	Twin-screw extrusion method	Sustained release with a rapid initial release of about 120 days Excellent stability with no signs of aggregation	[93]
PLGA microparticles	Ranibizumab	PLGA and PVA	Water-in-oil-in-water (W/O/W) double emulsion	Sustained release of mAB up to 3 weeks Suppression of VEGF-induced tube formation	[94]
Microspheres	Ranibizumab	PLGA, bovine serum albumin, PEG, Mg(OH)_2_, and PVA	Double emulsion, solvent evaporation technique	Reduction in lesions of choroidal neovascularization at lower doses No signs of retinal cellular dysfunction	[95]
Microsphere-hydrogel	Ranibizumab	PLGA, PVA, Bovine serum albumin (BSA), PEG, poly(ethylene glycol)-co-(L-lactic acid) diacrylate (PEG-PLLA-DA) and N-isopropylacrylamide (NIPAAm)	Modified double-emulsion, solvent evaporation technique, and free radical polymerization method	Increase in VPTT and decrease in swelling ratios with the increase in cross-linker concentration pH-sensitive controlled release of mAB for up to 6 months	[96]
Microsphere-hydrogel	Aflibercept	PLGA, PVA, BSA, PEG, PEG-PLLA-DA, and NIPAAm	Modified double-emulsion, solvent evaporation technique and free radical polymerization method	Controlled release of aflibercept ∙ No signs of toxicity from byproducts Retained bioactivity throughout the study No changes in the structure and functions of the retina	[97,98]
Polymeric nanoparticles	Aflibercept	PLGA and PVA	Double-emulsion diffusion method	Sustained release of drug for 7 days No signs of toxicity	{100}
Microparticles	PolyActive™	PEG, Polybutylphthalate	Water-in-oil-in-water emulsion (w/o/w) process	Sustained delivery of dAB for up to 6 months Protection of cynomolgus against laser-induced grade IV CNV	[100]
Intravitreal implants	Dexamethasone	PLGA and PCL	Extrusion method	High variability in the release profile of drugs shown by different methods and conditions	[101]

### 3.2. Delivery through Subretinal Space

**Figure 4:**
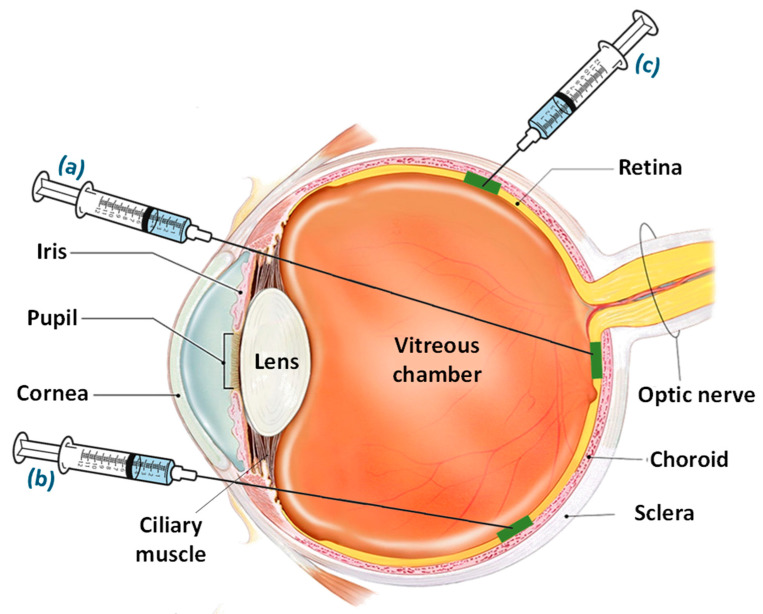
Approaches/routes for subretinal injection including (a) the transcorneal route entering through the pupil and passing through the lens, vitreous region, and retina, and (b) the transscleral route passing through the limbus region and vitreous, opposite to the retinal region into subretinal space, and (c) the transscleral route crossing through the Bruch’s membrane and choroid.

The region between the RPE layer and the photoreceptors is considered as a subretinal space which allows the direct delivery of drugs to the RPE and photoreceptor cells. The subretinal injections utilize trans-scleral and trans-corneal routes in animal studies for attaining the required desired concentration of a drug in subretinal space [113,114]. It involves the prior conducting of a vitrectomy to separate the posterior vitreous that may use acetonide triamcinolone for better visualization [114]. The subretinal injection primarily involves three approaches/routes for the administration of drugs: (a) the transcorneal route crossing through the pupil, lens, vitreous region, and retina; (b) the transscleral route through the limbus region and passing through the vitreous; and (c) the transscleral route crossing through the Bruch’s membrane and choroid [114]. Figure 4 illustrates the approaches for subretinal injection adopted by various researchers for the delivery of drugs for AMD. However, the subretinal delivery is associated with challenges due to high invasiveness and access to a small area upon every injection. Despite these obstacles, researchers have become interested in targeting the subretinal space for gene delivery and cell therapy [115]. Palucorcel is a newly developed cell-based therapy for AMD which contains human umbilical tissue-derived cells in a cryopreserved product [116]. A novel subretinal delivery injection of palucorel was evaluated for safety and efficacy in patients with GA. The treated patients showed mild and non-critical adverse events with no signs of retinal detachment or alterations in intraocular pressure. However, the palucorel was successfully delivered through the subretinal site, but the reduction in GA and improved visual acuity were not exhibited throughout the study [117].

Gene therapy is one of the effective approaches for dealing with AMD by incorporating healthy genes in the cells of patients to avoid or treat defective genetic pathways. The benefit of using gene therapy is that it provides long-lasting treatment and enables targeted ocular regions to generate their protective agents [118]. Streptococcus pyogenes Cas9 (SpCas9) mRNA targets VEGFa and Rho genes in RPE and photoreceptor cells by using SpCas9 or small nucleases in adeno-associated viruses. They are employed in the treatment of Leber congenital amaurosis type 10, which occurs due to mutations in the CEP290 gene [119]. A subretinal injection comprising SpCas9 mRNA and expression cassettes were found to be effective in wet-AMD. The modified lentiviruses inhibited VEGFa in RPE, resulting in a 63% reduction in choroidal neovascularization without affecting undesirable target edits and immune responses. This approach may also be suitable for other forms of retinal disorders wherein the restriction of neovascularization is required [120]. A lentiviral gene therapy vector (RetinoStat^®^) based on an equine infection-causing anemia virus was developed for delivering two anti-angiogenic genes (endostatin and angiostatin) to the retina to suppress angiogenesis and enhance the vision of patients [121]. RetinoStat^®^ was studied for subretinal delivery for the management of wet-AMD. The amount of endostatin and angiostatin increased after subretinal administration in rabbit eyes throughout the study. Ocular inflammation was reduced with 1 month of continuous dosing, with no considerable changes in electroretinograms and intraocular pressure [122]. The recombinant adeno-associated virus- (rAAV) based gene-therapy allows vector-like, soluble fms-like tyrosine kinase-1 (sFLT-1) to be delivered directly to the RPE and photoreceptor cells. This enables the uptake and transduction of viral vectors and sFLT-1 to express through protein-generating mechanisms of the cells [123]. A phase I trial of the subretinal injection of rAAV sFLT-1 demonstrated no proliferation in RPE cells, retinal scar production, or atrophic changes. However, some of the individuals encountered hemorrhages and cataract development. Overall, the product was well-tolerated and suitable for prolonged treatment for wet AMD [124]. Lambert et al. reported the investigation involving subretinal injections of adeno-associated virus-mediated gene therapy for targeting subretinal and outer retinal tissues with the cartilage oligomeric matrix protein angiopoietin-1 in mice simulated laser-assisted wet AMD. The results showed a reduction of about 29 to 33% in VEGF levels and 60 to 70% in the volume of choroid neovascularization. The vector-based product is appropriate for subretinal delivery and may serve as a promising treatment for neovascular AMD along with anti-VEGF agents [125].

### 3.3. Delivery through Suprachoroidal Space

The novel drug delivery techniques have facilitated better access to the suprachoroidal space for the treatment of ocular diseases. The drugs administered through the suprachoroidal space allow the attainment of higher concentrations in the retinal region, thereby reducing the undesirable delivery to the anterior ocular areas [126]. A novel antineoplastic agent called axitinib has a potent blocking activity over VEGF and platelet-derived growth factor (PDGF) receptors, therefore axitinib helps in the neovascularization and treatment of AMD [127]. The injectable suspension of axitinib (CLS-AX) was designed as a long-acting preparation for neovascular AMD. The ocular distribution demonstrated higher localization of axitinib in the sclera, RPE, and choroid, followed by the retina and vitreous. The product showed a marked reduction in the eye lesions in the rats, good tolerability, and no signs of toxicity [128]. Hancock et al. studied the bioavailability and sustainability of the small molecule suspension of A01017 (complement factor D inhibitor). The suprachoroidal injection was tolerated adequately in rabbits with minimum signs of toxicity. The suspension showed high sustained exposure of A01017 to the RPE, choroid, and sclera, along with first-order elimination throughout the 92-day study period [129].

The suprachoroidal graft of autologous cells was also proposed as a treatment for dry AMD, owing to its impact on the enhancement of visual acuity and microperimetric responses. The technique involved the implantation of adipose stem cells in the suprachoroidal space to stimulate the secretion of growth factors. The outcome was a significant improvement in visual acuity after six months, along with the maintenance of growth factor secretion and choroidal flow [130]. The restoration effect of grafted autologous cells on retinal cells was investigated to assess the continuous secretion of growth factors in patients with dry AMD. The best corrected visual acuity was found to be significantly improved in patients with higher retinal thickness averages due to the availability of greater cellularity [131].

Implantation containing adipose tissue-derived mesenchymal stem cells was evaluated for its safety and efficiency in patients with dry AMD. There were no occurrences of systemic or ocular complications in any of the patients with improvement in the visual field, visual acuity, and mf-ERG recordings [132]. Zhang et al. assessed the intraocular cell technology-based implant containing ciliary neurotrophic factor for the management of GA. The thickness of the retina increased with the incorporation of treatment in a dose-dependent manner, subsequently stabilizing the visual acuity. The implant delivered with newer technology was adequately tolerable by the patients and retarded the sequences of vision loss in GA [133]. The biodegradable nanoparticles can be used for the delivery of the VEGF-binding protein expression plasmid to RPE or even the entire eye for a longer duration through suprachoroidal injection. The anti-VEGF activity of nanoparticles was indicated through the repression of vascular leakage and neovascularization. The therapeutic benefits were further displayed by a considerable rise in sFlt1 retinal protein upon 1 month of therapy [134]. Patel et al. reported that the suprachoroidal delivery of aflibercept showed a reduction of the neovascular area in laser-induced neovascularized rat models. The treated animals showed a marked reduced neovascular leak area from 4862 pixels2 to 3318 pixels2 during the 21-day study period. The researchers suggest that suprachoroidal injection showed promise for the delivery of other anti-VEGF agents, especially in cases of wet AMD [135].

### 3.4. Port Delivery System

A port delivery system (PDS) encompasses a robust reservoir fabricated for the prolonged delivery of the drug into the vitreous cavity after being implanted. The ability of PDS to release the anti-VEGF medication for a longer duration helps in reducing the overdependency on intravitreal injections for the treatment of AMD. PDS is applied through the surgical insertion of the device into the scleral space through conjunctival peritomy. After implantation, the drug diffuses from the release control element in a sustained manner into the vitreous, which can be filled again once emptied [136]. A phase 2 trial of PDS of ranibizumab (10 to 100 mg/mL) was conducted for assessing its safety and efficacy. PDS implant insertion and refilling procedures were endured by the patients with a reduction in postoperative vitreous hemorrhage rate to 4.5% and no signs of implant clogging. Furthermore, the vision and structural outcomes after 9 months of administration of PDS (100 mg/mL) were similar to that of intravitreal ranibizumab (0.5-mg injection) [76]. Wykoff et al. discussed the pharmacokinetic outcomes of PDS of ranibizumab measured in the samples collected from serum and aqueous humor of the patients. The median serum concentrations of ranibizumab for PDS 10 mg/mL were found to be lower than the serum concentration achieved from intravitreal ranibizumab (0.5-mg injection). On the other hand, the median serum concentration resulting from 40 mg/mL and 100 mg/mL PDS were within the range of monthly intravitreal 0.5-mg injection throughout the 12-month study period [137]. Apart from this, the PDS of ranibizumab is also commercially viable owing to the sustained delivery of the drug, which reduces the dosing frequency. This results in lowering the cost of treatment, thereby reducing the burden of the treatment to both the patient and the health care system [138]. A phase 3 study of 24-week dosing with PDS ranibizumab is undergoing for evaluating its safety and efficacy in comparison with monthly intravitreal injections of ranibizumab. The constant ranibizumab delivery with PDS (refill after 6 months) demonstrated efficacy comparable to the intravitreal injections with more than 98% of the patient not requiring any supplemental treatment as observed for the first 6 months [139].

### 3.5. Delivery through Other Routes

Several studies have explored the utilization of some of the less preferred routes of administration to combat the limitations associated with existing therapies. Some of the routes reported in recent times chosen for drug delivery for AMD are the subconjunctival, topical, oral, etc. The subconjunctival region is located under the conjunctival membrane covering the sclera. The subconjunctival space is mostly chosen for delivering drugs to anterior ocular regions [140]. A depot formulation of sirolimus (mTOR inhibitor) was designed to be administered as a subconjunctival injection for the treatment of GA. The drug in its carrier formulation is well-tolerated in patients without any significant indications of adverse reactions. However, the outcomes of the study were not favorable concerning the structural and functional impact of therapy. There was an evident increment in GA areas and receding visual acuity in individuals after 24 months. Furthermore, there were no significant differences in the retinal thickness, drusen area, and macular sensitivity over 24 months [141]. Chaw et al. developed liposomal nanocarriers meant for subconjunctival administration and assessed their in vivo biodistribution using fiberoptic Confocal Laser Microendoscopy and radiotracing. Large positively charged liposomes demonstrated retention around the injection site for a longer duration, while neutral/negative small-sized liposomes showed better distribution in the limbus region. The nanocarriers can be optimized for encapsulation and controlled delivery of the drugs and biologicals used in AMD treatment [142].

Numerous efforts were also made to deliver drugs and biologicals through the topical route owing to convenient and non-invasive administration [143]. A topical product (PAN-90806) which inhibits the tyrosine kinase inhibitor of VEGF-A and Platelet-derived growth factor (PDGF) was subjected to a Phase I/II trial. According to the results, about 50% of the patients under treatment did not need any rescue therapy, and more than 80% showed improvement [144]. Danis et al. reported the development of another VEGF-A and PDGF inhibitor topical formulation (Pazopanib). A randomized trial has been conducted for examining their safety and efficacy in the treatment of wet AMD. The outcomes showed that there was no considerable reduction in retinal thickness, except for those having the CFH-TT genotype and receiving treatment three times daily (5 mg/mL) [145]. The Pazopanib eye drops in combination with ranibizumab did not show any therapeutic superiority over existing ranibizumab therapy. Consequently, the development of the product has been stopped [146]. Cogan et al. designed topical formulations of ranibizumab and bevacizumab delivered using cell-penetrating peptides (CPPs). The in vitro studies showed no toxicity from CPPs. The clinically requisite concentrations of CPPs along with anti-VEGF agents were detected in the posterior region of the rat eye. The efficiency of a daily administered topical agent in decreasing the choroidal neovascularization was comparable to the intravitreal injection of anti-VEGF agents [147].

Although the oral delivery of drugs provides a means of convenient and non-invasive administration, it is rarely chosen for the treatment of AMD due to the presence of ocular barriers, preventing effective delivery to the posterior regions. The phase II trial of an oral tablet formulation (X-82) containing anti-VEGF/PDGF agents was conducted to examine the efficacy of wet AMD. Although the product showed comparable improvement in visual acuity at higher doses, the limitations in safety and tolerability of the formulation resulted from the halt in further development. A case-control study reported the correlation between the oral administration of metformin and a reduction in the chances of developing AMD. The assessments indicate that metformin may possess therapeutic potential for AMD. However, there is a need for a comprehensive clinical study to assert the benefits of oral metformin therapy in preventing AMD development [148]. Stewart et al. reported a multicenter Phase 2a pilot clinical trial conducted over an orally administered product (AKST4290) for AMD. AKST4290 inhibits C-C Motif Chemokine Receptor 3 (CCR3), an eotaxin receptor that suppresses inflammation and neovascularization for the management of wet AMD. The study showed a substantial enhancement in the visual acuity of the patients with no indications of severe adverse events. It was suggested that further studies will be carried out as randomized controlled trials with a placebo in order to be assured about the safety of the product [149].

## 4. Overview of Patent Situation

AMD management involves continuous observation, regular follow-up, and documentation for timely recognition of visual function, or else the condition leads to mortality. Several studies reported on the management of AMD, as presented in the previous section, both with the safety and efficacy of formulations alone and in combination with treatments such as photodynamic therapy. Practical therapeutics strategies for a complex disease such as AMD require the combination of multiple factors, including diet, lifestyle, and improved pharmacological interventions, and the direct development of not only effective but also safe treatment strategies. The systemic quality, safety, and efficacy of many investigated products after successful clinical trials leads to the granting of such patents, indicating the extreme necessity of these products [150]. Besides several synthetic medications alone and in combination that have been effectively used, other therapeutics such as endothelial growth factors in sustained release form with gene therapy have also been reported in the management of AMD, signifying the requirement for a variation in therapeutics [78]. Several companies and institutions have filed patents, and the number of patents filed was particularly higher in the USA as compared to other countries. Patents were based on either the delivery of novel drugs/biologicals or employing novel strategies for the efficient delivery of existing therapeutic agents. The use of synthetic drugs, recombinant vectors, genetic variants, monoclonal antibodies, fusion proteins, expression vectors, etc. were some of the major approaches. However, the common target for the majority of researchers involved targeted suppression of the VEGF system, but some of them also discussed reducing the inflammatory response, prophylactic control, photosensitizing therapy, and preventing retinal degeneration, etc.

This section is included to provide an outline of the patents granted or applied in recent years related to the use of novel delivery approaches for the treatment of AMD. A recent US patent application of mesozeaxanthin for the management of macular diseases, specifically AMD, proposed that the supplementation of mesozeaxanthin (dosage 0.5 and 50 mg/day) either alone or in combination with other carotenoids and vitamins may increase macular pigments [151]. Another patent by Marcus et. al., disclosed the design and synthesis of a prodrug comprising a therapeutic agent associated with carotenoids including anecortave acetate, anti-VEGA aptamer, or protein kinase C inhibitor linked to carotenoids for the management of macular and retinal diseases [152]. Furthermore, an additional US patent disclosed a novel expression cloning strategy known as CHANGE (Comparative Hybridization Analysis of Gene Expression) that indicated a 5.5-fold increment in MT1-MMP mRNA levels in the retina affected with AMD compared with control eyes. Moreover, the discloser also elaborated the method for the treatment of AMD by targeting either MT1-MMP nucleic acid or protein [153]. Furthermore, the US patent registered by Tamaki et al., disclosed the use of vaccine therapy for the treatment of CNV utilizing VEGFR2-derived peptides (VIAMFFWLL) as an immunizing agent for the treatment or prevention of CNV by suppressing VEGF upregulation [154].

Expert opinion on the patent registered by Borodic for the administration of botulinum-toxin as an extra ocular infusion avoiding the risk of direct intraocular injections and complications associated with the patent indicated that co-administration with proteins such as hemagglutinin and monoclonal antibody makes a novel system for the development of the combined approach in the management of AMD [155]. Moreover, the summarized patent distribution with regards to the year of filing the patent, jurisdiction, applicant, and details of the invention is presented in Table 3.

## 5. Conclusions

Ocular barriers pose challenges in the treatment of AMD, leading to therapies with suboptimal efficacy. To overcome this, there is a requirement for the development of novel therapies and drug delivery strategies to fulfill the unmet need. This review was focused on discussing drug delivery in AMD concerning currently available therapeutic approaches, emerging therapies, and the situation of patents. The intravitreal space is the most targeted route for AMD treatment for the delivery of anti-VEGF agents such as bevacizumab, ranibizumab, aflibercept, etc. The studies have also reported the use of novel delivery systems such as polymeric nanoparticles, liposomes, implants, microspheres, etc., which could help in enhancing therapeutic efficiency. The efforts were mostly towards controlling/sustaining release, prolonging residence time, reducing the incidence of adverse events, improving stability, etc. Subretinal and suprachoroidal routes were also investigated for cell-based and gene therapies for dry and neovascular AMD. The PDS of Ranibizumab is another interesting prospect for sustained delivery of the therapeutic agents, allowing for the reduction in dosing frequency. Lastly, the current patent situation included in the review showed that the suppression of the VEGF system is the major target for the treatment of AMD.

## Figures and Tables

**Table 3:** Patents published recently based on the use of novel delivery strategies for the treatment of AMD.

Patent Number (Publication Year)	Jurisdiction	Applicant	Title	Detail of Work	Ref.
WO0023262W (2014) CA2940513A (2015) US10981961B2 (2021)	WIPO, Canada, & United States	University of Florida	Delivery of card protein as therapy for ocular inflammation	Methods of administration of an expression vector that delivers a secretable and cell penetrating CARD to a subject in need of treatment or the prevention of AMD or related conditions	[156,157,158]
US0064403W (2017)	United States	Regeneron Pharmaceuticals, Inc.	Methods of associating genetic variants with a clinical outcome in patients suffering from AMD treated with anti-VEGF	Associating a genetic variant with intraretinal fluid as a marker for response to therapy with anti-VEGF in AMD with regard to visual acuity, anatomic outcomes or treatment frequency	[159]
CA 3044385A (2018) CN80074187A (2019) JP043133W (2018)	Canada, China, & Japan	Santen Pharma Co Ltd.	Method of predicting responsiveness of wet AMD patient to anti-VEGF therapy	Predicting the efficacy of an anti-VEGF drug treatment for exudative AMD by measuring the concentration of at least one marker protein and correlating the concentration measured with the efficacy of the anti-VEGF drug	[160,161,162]
CA3076905A (2019) AU250797A (2018) US16645877A (2020)	Canada, Australia, & United States	Regeneron Pharmaceuticals, Inc.	Treatment of ocular diseases with fully-human post-translationally modified Anti-VEGF Fab	Compositions and approaches for the delivery of a fully human post-translationally modified (HuPTM) monoclonal antibody (mAb) or the antigen-binding fragment of a mAb against human vascular endothelial growth factor (hVEGF) to the retina/vitreal humour in the eye(s) of human subjects diagnosed with ocular diseases	[163,164,165]
US16501058A A1(2020)	United States	Henry J. Smith	Treatment of age-related macular degeneration	Novel strategy for the treatment of vascular and inflammatory aspects of the AMD using a nanocarrier coated with an anti-VEGFR targeting agent.	[166]
KR018859W (2022)	South Korea	BenoBio Co., Ltd.	Novel peptide inhibiting BET protein and composition comprising same for prophylaxis and treatment of eye disease	Novel peptide contained in a specialized formulation to ameliorate inflammation caused by retinal damage resulting from epigenetic changes, and effects of the peptide on the prophylaxis and amelioration of various eye diseases caused by retinal degeneration.	[167]
CN10705207A (2019)	China	Chengdu Genevector Biotechnology Co., Ltd.	Fusion protein, virus vector and medicine for treating age-related macular degeneration	Fusion protein, virus vector and medicine for treating age-related macular degeneration and relationship to the technical field of gene therapy. The fusion protein can treat the age-related macular degeneration, and has the characteristics of being low in dosage, continuous in action, and low in immunization risk	[168]
KR20180028890A (2017) EP17848931A (2019) AU323898A (2021) US16327850A (2020)	South Korea, Europe, Australia, and United States	Soonchunhyang University Industry Academy Cooperation Foundation; CuroGene Life Sciences Co., Ltd.	Pharmaceutical composition containing mTOR inhibitor for treating macular degeneration	Formulation designed for the management of AMD using a recombinant vector into which an shRNA having mTOR inhibition was incorporated. The pharmaceutical composition according to the present invention can effectively treat age-related macular degeneration, a representative retinal disease that causes blindness in adults.	[169,170,171]
US16987831A (2020)	United States	Gary E. Borodic	Novel method of treating macular degeneration using botulinum toxin-based pharmaceuticals	Methods of treatment for prevention and/or treatment of visual loss from AMD by employing formulations including botulinum neurotoxin. The formulations may be applied to an intraocular or extraocular region of a patient for altering VEGF production and is associated with the suppression of chorio-retinal leakage from macular pathologies.	[172]
US 202017611972A (2022)	United States	The Research Institute at Nationwide Children’s Hospital	Improved delivery of gene therapy vectors to retinal cells using a glycoside hydrolase enzyme	Methods of targeting specific cell types within the retina using optimized gene therapy vectors in combination with a glycoside hydrolase enzyme, such as neuraminidase for treating visual impairment, retinal degeneration, and vision-related disorders	[173]
EP08827362A (2014)	Europe	Santen Pharmaceutical Co., Ltd.	Rapamycin formulations for treatment of age-related macular degeneration	Liquid formulations for the treatment and prevention of AMD progression by delivery of rapamycin (sirolimus) to the eye of the human subject	[174]
US74348908A (2015)	United States	CeramOptec Industries Inc.	PEGylated compounds for age-related macular degeneration	PEGylated liposome containing a hydrophobic photosensitizer and the photodynamic therapy method for treating CNV associated with AMD	[175]
KR20180112734A (2019)	South Korea	CuroGene Life Sciences Co., Ltd.	Pharmaceutical composition for treating macular degeneration containing AAV including cDNA of the soluble VEGFR variant	Pharmaceutical product for treating or preventing AMD to a recombinant vector carrying a soluble VEGF receptor variant cDNA	[176]

## Data Availability

Not applicable.
